# Maternal diabetes and risk of offspring congenital heart diseases: the Japan Environment and Children’s Study

**DOI:** 10.1265/ehpm.23-00358

**Published:** 2024-04-05

**Authors:** Maiko Nagasawa, Satoyo Ikehara, Yuri Aochi, Kanami Tanigawa, Tetsuhisa Kitamura, Tomotaka Sobue, Hiroyasu Iso, the Japan Environment and Children’s Study (JECS) Group

**Affiliations:** 1 Division of Environmental Medicine and Population Sciences, Department of Social and Environmental Medicine, Graduate School of Medicine, Osaka University; 2 Osaka Regional Center for Japan Environment and Children’s Study (JECS), Osaka University; 3 Institute for Global Health Policy Research, Bureau of International Health Cooperation, National Center for Global Health and Medicine; 4 Osaka Maternal and Child Health Information Center, Osaka Women’s and Children’s Hospital, Osaka, Japan

**Keywords:** Maternal diabetes, Pre-pregnancy diabetes, Gestational diabetes, Congenital heart diseases, Cohort studies

## Abstract

**Background:**

Few prospective cohort studies have examined the association between maternal diabetes, including pre-pregnancy and gestational diabetes, and the risk of congenital heart disease (CHD) in Asian offspring.

**Methods:**

We examined the association between maternal diabetes and offspring CHD among 97,094 mother-singleton infant pairs in the Japan Environment and Children’s Study (JECS) between January 2011 and March 2014. Odds ratios (OR) and 95% confidence intervals (CI) of offspring CHD based on maternal diabetes (pre-pregnancy diabetes and gestational diabetes) were estimated using logistic regression after adjusting for maternal age at delivery, pre-pregnancy body mass index (BMI), maternal smoking habits, alcohol consumption, annual household income, and maternal education. The diagnosis of CHD in the offspring was ascertained from the transcript of medical records.

**Results:**

The incidence of CHD in the offspring was 1,132. Maternal diabetes, including both pre-pregnancy diabetes and gestational diabetes, was associated with a higher risk of offspring CHD: multivariable OR (95%CI) = 1.81 (1.40–2.33) for maternal diabetes, 2.39 (1.05–5.42) for pre-pregnancy diabetes and 1.77 (1.36–2.30) for gestational diabetes. A higher risk of offspring CHD was observed in pre-pregnancy BMI ≥25.0 kg/m^2^ (OR = 2.55, 95% CI: 1.74–3.75) than in pre-pregnancy BMI <25.0 kg/m^2^ (OR = 1.49, 95% CI: 1.05–2.10, p for interaction = 0.04).

**Conclusions:**

Maternal diabetes, including both pre-pregnancy and gestational, was associated with an increased risk of CHD in offspring.

## Introduction

Congenital heart disease (CHD) is the most common congenital disorder among newborns [1–4]. Currently, more than 90% of children born with CHD may reach the age of 18 years owing to revolutionary surgical and medical care [5, 6]. However, the survival rate in adults with severe CHD is significantly lower than that in the general population [7, 8]. The increasing trend of CHD has led to an increasing prevalence of CHD in Asia compared with other regions [3, 9].

A recent meta-analysis of 26 cohort studies showed a significantly increased risk of CHD in the offspring of women with maternal diabetes than those without diabetes [10]. Maternal diabetes consists of pre-existing diabetes mellitus during pregnancy and gestational diabetes mellitus (GDM) [11, 12]. Pre-existing diabetes mellitus during pregnancy, which is referred to in this article as pre-pregnancy diabetes, is glucose intolerance diagnosed before pregnancy [13]. GDM is defined as a glucose metabolism disorder of both insulin resistance and hyperglycemia [14] with onset or first recognition during pregnancy [15, 16]. The prevalence of diabetes mellitus among women aged 20–39 years is low in Japan; the prevalence of diabetes strongly suspected was 1.9% according to the National Health and Nutrition Survey Japan in 2019 [17]. The estimated prevalence of GDM in Japanese pregnant women based on the criteria issued by the Japan Diabetes Society was 2.4–6.6% [18]. Thus, GDM may significantly impact the health of children. The harmful effect of maternal diabetes on CHD in offspring has been reported in Western countries and China, however, it has not been investigated in other Asian countries.

Therefore, our novel study examined the associations between maternal diabetes, including pre-pregnancy diabetes and GDM, and the risk of offspring CHD among 97,094 mother-singleton infant pairs using data from a large birth cohort study.

## Methods

This prospective cohort study used data from the Japan Environment and Children’s Study (JECS), an ongoing, nationwide government-funded birth cohort study designed to investigate the environmental factors that affect children’s development and health during the fetal period and/or in early childhood [19, 20]. Recruitment of pregnant women and their partners was conducted between January 2011 and March 2014, and 104,062 fetal records were registered. For the JECS, 15 Regional Centers were selected to cover wide geographical areas from urban to rural areas in Japan: Hokkaido, Miyagi, Fukushima, Chiba, Kanagawa, Koshin, Toyama, Aichi, Kyoto, Osaka, Hyogo, Tottori, Kochi, Fukuoka, and Southern Kyushu/Okinawa. The eligibility criteria for the participants were as follows: 1) living in the study area at the time of enrollment and expected to continue to live in Japan for the foreseeable future, 2) expected delivery date was between August 2011 and mid-2014, and 3) capable to participate in the study without difficulty (i.e. had adequate Japanese language comprehension to completely respond to self-administered questionnaire) [21]. Previous studies have described the details of the JECS project [21, 22]. This study used the datasets jecs-ta-20190930 and jecs-qa-20210401.

**Fig. 1:**
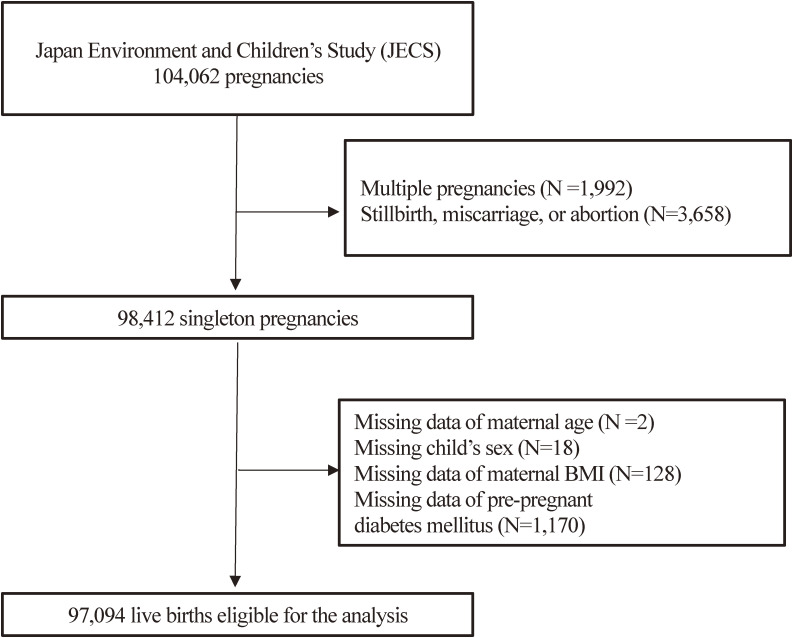
Flowchart of the process of selecting participants for analysis.

There were 104,062 fetal records in the JECS. Of these, we excluded multiple pregnancies (N = 1,992) and stillbirths, miscarriages, or abortions (N = 3,658). Among the 98,412 singleton live births, the following data were missing: maternal age in two pregnant women, the sex of the child in 18, maternal body mass index (BMI) in 128, and data for pre-pregnancy diabetes in 1,170; therefore, we excluded them. Finally, 97,094 mother-singleton infant pairs who completed the JECS questionnaire were included in the current analysis. (Fig. 1)

Information on women with pre-pregnancy diabetes was defined based on self-reported diagnosis at the first trimester questionnaire. Women with GDM were identified from medical records at the time of delivery, and physicians, midwives, nurses, and/or research coordinators performed the transcription [23]. Patients with GDM were diagnosed based on the results of a 75 g oral glucose tolerance test (75 g OGTT) according to the criteria of the Japan Diabetes Society [14]. The procedures of GDM diagnosis are as follows: As a screening test for GDM, random blood glucose tests for all the pregnant women in the first and second trimester was performed. When the random blood glucose levels were 100 mg/dL (5.5 mmol/L) or more, then a 75 g OGTT was performed for further diagnosis. The GDM was diagnosed when one or more of the following criteria met in a 75 g OGTT; 1) fasting plasma glucose level ≥ 92 mg/dL (5.1 mmol/L), 2) 1-hour plasma glucose level ≥ 180 mg/dL (10.0 mmol/L), and/or 3) 2-hour plasma glucose level ≥ 153 mg/dL (8.5 mmol/L). Women with diabetes mellitus before pregnancy were precluded from GDM screening test [16].

The outcome was offspring CHD, which was diagnosed by an obstetrician or pediatrician. We used data on offspring CHD detected during the newborn or one-month-old periods, which was confirmed by medical information from the hospital.

### Statistical analyses

We compared the distribution of characteristics based on the diagnosis of maternal diabetes, which was categorized as pre-pregnancy diabetes and GDM. Odds ratios (OR) and 95% confidence intervals (CI) for offspring CHD based on maternal diabetes were estimated by logistic regression. The same analyses were conducted to estimate the OR for offspring CHD based on pre-pregnancy diabetes and GDM. We adjusted for selected potential confounding factors, including maternal age at delivery (continuous), pre-pregnancy body mass index (BMI) (<18.5 kg/m^2^. 18.5 to <25.0 kg/m^2^, 25.0 to <27.0 kg/m^2^, or ≥27.0 kg/m^2^), maternal smoking habits at the first trimester (never, past, quit smoking after realizing pregnancy, or current smokers), maternal alcohol consumption at the first trimester (never, past, or current drinkers), annual household income (<4,000,000 JPY, 4,000,000–5,999,999 JPY, 6,000,000–7,999,999 JPY, 8,000,000–9,999,999 JPY, or ≥10,000,000 JPY), and maternal highest level of education (high school or less, vocational school, junior college, or university or more). BMI was calculated as the weight (kg) divided by the square of height (m^2^). The effect modification was examined with maternal age at delivery (<35 years / ≥35 years), pre-pregnancy BMI (<25.0 kg/m^2^ / ≥25.0 kg/m^2^), smoking habits (never / past (past or quit) or current smokers), alcohol consumption (never / past or current drinkers), annual household income (<4,000,000 JPY / ≥4,000,000 JPY), and maternal education (high school or less/ vocational school or junior college or more), by using the cross-product of maternal diabetes and these variables. We categorized the missing covariates as dummy variables and included in the model of multiple logistic regression analyses.

Two-tailed probability values of 0.05 were considered statistically significant. Statistical analyses were performed using the SAS software (version 9.4; SAS Institute, Cary, NC, USA).

## Results

Among 97,094 singleton pregnant women, 3,050 women (3.1%) had maternal diabetes with the breakdown of 199 pre-pregnancy diabetes (0.2%), and 2,851 GDM (2.9%). Considering the newborn and one-month-old groups, 1,132 infants (1.2%) were diagnosed with CHD.

Table 1 reveals the characteristics of the participants based on the categories of non-diabetes and maternal diabetes (pre-pregnancy diabetes or GDM). Compared with women without diabetes, those with pre-pregnancy diabetes were more likely to be older, and to have a higher pre-pregnancy BMI, have past or current smoking habits, consume less alcohol, have lower annual household income, and have less education. Women with GDM had similar characteristics to those with pre-pregnancy diabetes except for the higher annual household income than those without diabetes.

**Table 1:** Characteristics of study participants according to pre-pregnancy diabetes and GDM.

	**Non-diabetes**		**Maternal diabetes**			

			**Pre-pregnancy diabetes**		**GDM**	
Number of subjects	94,044		199		2,851	
Maternal age, year (SD)	31.1	(5.0)	32.0	(5.0)	33.4	(5.0)
Pre-pregnancy BMI, n (%)						
<18.5	15,399	(16.4)	13	(6.5)	273	(9.6)
18.5–25.0	69,325	(73.7)	97	(48.7)	1,663	(58.3)
25.0–27.0	4,258	(4.5)	13	(6.5)	249	(8.7)
≥27.0	5,062	(5.4)	76	(38.2)	666	(23.4)
Smoking habits, n (%)						
Never-smokers	54,481	(57.9)	92	(46.2)	1,547	(54.3)
Past-smokers	22,003	(23.4)	63	(31.7)	757	(26.6)
Quit smoking after pregnant	12,370	(13.2)	30	(15.1)	372	(13.0)
Current-smokers	4,492	(4.8)	13	(6.5)	152	(5.3)
Missing	698	(0.7)	1	(0.5)	23	(0.8)
Alcohol consumption, n (%)						
Never-drinkers	32,319	(34.4)	89	(44.7)	1,062	(37.3)
Past-drinkers	51,958	(55.3)	101	(50.8)	1,516	(53.2)
Current-drinkers	9,337	(9.9)	8	(4.0)	257	(9.0)
Missing	430	(0.5)	1	(0.5)	16	(0.6)
Annual household income, n (%)						
<4,000,000 JPY	34,867	(37.1)	93	(46.7)	1,019	(35.7)
4,000,000–5,999,999 JPY	28,602	(30.4)	58	(29.1)	888	(31.1)
6,000,000–7,999,999 JPY	13,766	(14.6)	20	(10.1)	433	(15.2)
8,000,000–9,999,999 JPY	5,641	(6.0)	5	(2.5)	174	(6.1)
≥10,000,000 JPY	3,680	(3.9)	3	(1.5)	135	(4.7)
Missing	7,488	(8.0)	20	(10.1)	202	(7.1)
Maternal highest level of education, n (%)						
High school or less	33,576	(35.7)	101	(50.8)	1,042	(36.5)
Vocational school	21,168	(22.5)	47	(23.6)	651	(22.8)
Junior college	17,764	(18.9)	30	(15.1)	551	(19.3)
University or more	20,121	(21.4)	20	(10.1)	564	(19.8)
Missing	1,415	(1.5)	1	(0.5)	43	(1.5)

Table 2 indicates the ORs (95% CI) for offspring CHD based on maternal diabetes, pre-pregnancy diabetes, and GDM, respectively. Women with maternal diabetes had an increased risk of offspring CHD than women without maternal diabetes. The multivariable OR was 1.81 (95% CI: 1.40–2.33) after adjustment for maternal age at delivery, pre-pregnancy BMI, smoking habits, alcohol consumption, annual household income, and maternal education. Both pre-pregnancy diabetes and GDM had an increased risk of offspring CHD: the corresponding multivariable ORs (95% CI) were 2.39 (95% CI: 1.05–5.42) and 1.77 (95% CI: 1.36–2.30). On testing the effect modification by maternal age, pre-pregnancy BMI, smoking habits, alcohol consumption, annual household income, and maternal education, a higher risk was observed in pre-pregnancy BMI ≥25.0 kg/m^2^ (OR = 2.55, 95% CI: 1.74–3.75) than in pre-pregnancy BMI <25 kg/m^2^ (OR = 1.49, 95% CI: 1.05–2.10) (p for interaction = 0.04). No effect modification by maternal age, smoking habits, alcohol consumption, annual household income, or maternal education was observed (Table 3).

**Table 2:** Odds ratios for offspring CHD according to maternal diabetes, pre-pregnancy diabetes and GDM.

	**Non-diabetes**	**Maternal diabetes**	**Pre-pregnancy diabetes**	**GDM**
Number of subjects	94,044	3,050	199	2,851
Cases of CHD	1,063	69	6	63
Incidence of CHD (%)	1.13	2.26	3.02	2.21
Crude OR (95% CI)	1.00	2.03 (1.58–2.59)	2.72 (1.20–6.14)	1.98 (1.53–2.56)
Adjusted OR (95% CI)	1.00	1.81 (1.40–2.33)	2.39 (1.05–5.42)	1.77 (1.36–2.30)

Adjusted for maternal age, pre-pregnancy BMI, smoking habits, alcohol consumption, annual household income, and maternal education.

**Table 3:** Association between maternal diabetes and offspring CHD stratified by potential confounding factors.

	**Non-diabetes**	**Maternal diabetes**	***p for interaction***
Age <35			0.33
Cases/N	759/69,132	33/1,721	
Adjusted OR (95% CI)	1.00	1.68 (1.18–2.40)	
Age ≥35			
Cases/N	304/24,912	36/1,329	
Adjusted OR (95% CI)	1.00	2.01 (1.40–2.88)	

BMI <25.0			0.04
Cases/N	936/84,724	34/2,046	
Adjusted OR (95% CI)	1.00	1.49 (1.05–2.10)	
BMI ≥25.0			
Cases/N	127/9,320	35/1,004	
Adjusted OR (95% CI)	1.00	2.55 (1.74–3.75)	

Never smokers			0.44
Cases/N	612/54,481	40/1,639	
Adjusted OR (95% CI)	1.00	1.99 (1.43–2.77)	
Past or current smokers			
Cases/N	441/38,865	29/1,387	
Adjusted OR (95% CI)	1.00	1.72 (1.16–2.53)	

Never drinkers			0.87
Cases/N	1,123/33,059	28/411	
Adjusted OR (95% CI)	1.00	2.03 (1.37–3.03)	
Past or current drinkers			
Cases/N	1,841/62,459	41/718	
Adjusted OR (95% CI)	1.00	1.77 (1.30–2.45)	

Annual household income <4,000,000 JPY	0.65		0.65
Cases/N	718/63,449	44/2,058	
Adjusted OR (95% CI)	1.00	1.74 (1.27–2.38)	
Annual household income ≥4,000,000 JPY			
Cases/N	255/23,087	18/770	
Adjusted OR (95% CI)	1.00	1.98 (1.21–3.24)	

Maternal education: High school or less			0.17
Cases/N	382/33,576	21/1,143	
Adjusted OR (95% CI)	1.00	1.46 (0.93–2.30)	
Maternal education: Vocational school or junior college or more			
Cases/N	648/59,053	46/1,863	
Adjusted OR (95% CI)	1.00	2.10 (1.54–2.85)	

Adjusted for maternal age, pre-pregnancy BMI, smoking habits, alcohol consumption, annual household income, and maternal education except for stratified variables.

## Discussion

In this large prospective cohort study, we observed a positive association between maternal diabetes including pre-pregnancy diabetes and GDM, and the risk of CHD in the offspring. A positive association was more evident for pre-pregnancy diabetes than for GDM, albeit in a small number of cases.

Previous studies have reported a similar association between maternal diabetes including pre-pregnancy diabetes or GDM, and the risk of offspring CHD [11, 24, 25]. A large population-based study using health care records in China reported the association between maternal diabetes and the risk of offspring CHD (OR = 1.80, 95% CI: 1.31–2.46) [25]. Another large population-based study of 48,249 patients with CHD using the data from the Texas Birth Defects Registry and state-wide vital records for deliveries showed the association with offspring CHD even after adjustment for race/ethnicity (34.0% Whites, 10.8% Black, 52.3% Hispanic, 2.9% others) for maternal diabetes (PR = 1.93, 95% CI: 1.84–2.03), for pre-pregnancy diabetes (PR = 3.24, 95% CI: 2.86–3.67), and for GDM (PR = 1.49, 95% CI: 1.39–1.60) [26].

The mechanisms for maternal glucose metabolism and offspring CHD are described as follows. Fetal heart development is complete by the 9th week of gestation [27]. Maternal diabetes, especially pre-pregnancy diabetes, has been reported as a risk factor for CHD because high glucose levels during organogenesis cause cardiovascular malformations through complex and multifactorial fetal molecular responses [28, 29]. For example, changes in the signaling pathways regulating insulin sensitivity may affect embryogenesis and fetal development [30]. Enhanced oxidative stress directly causes DNA damage and affects cardiac development [28].

The association with the risk of offspring CHD was weaker for GDM than maternal diabetes or pre-pregnancy diabetes probably because GDM is usually diagnosed around 24–28 weeks which was far later than the completion of fetal heart development. The potential effect of GDM on the offspring CHD risk was through accompanied hyperglycemia or diabetes at the early gestation.

Our results indicated that maternal diabetes with BMI ≥25.0 kg/m^2^ was associated with a higher risk of offspring CHD than that with BMI ≥25.0 kg/m^2^. As pre-pregnancy obesity is associated with an increased risk of gestational diabetes, a few of the effects of diabetes on offspring CHD may be mediated by impaired glucose regulation when the maternal BMI is high. In addition, obesity and diabetes promote metabolic changes in lipids, carbohydrates, insulin resistance [31, 32], altered activity of adipocyte hormones [32], disrupted micronutrient metabolism, and elevated oxidative stress [10, 33]. The effects of these nutritional and/or chemical changes during pregnancy may alter the intrauterine environment and fetal developmental pathways [10].

The recent increasing trend for the prevalence of GDM is more pronounced compared with the trend for the prevalence of pre-pregnancy diabetes [34]. In the United States, approximately 90% of maternal diabetes was GDM, whereas pre-pregnancy diabetes was only 10% [35]. Therefore, GDM has a greater impact on maternal and child health than pre-pregnancy diabetes. The assessment of glycemic levels in early pregnancy may be important for the early detection of abnormal glucose tolerance.

The main strengths of our study are its large sample size and prospective cohort design. This is the first report to investigate the association between maternal diabetes and offspring CHD in Japan. However, we could not evaluate the potential effect of blood glucose control status on the risk of CHD because of the lack of data. Furthermore, we did not examine the association with the risk of CHD phenotype because we collected the data as the broad category of “congenital heart disease.” [1]. According to a previous study, ventricular septal defect, atrial septal defect, and patent ductus arteriosus, the most frequent and mild types of CHD, consisted of approximately 60% of total CHDs, while the severe type of CHD such as pulmonary stenosis and tetralogy of Fallot did about 10% [9].

## Conclusions

Our study is the first to provide evidence of the association between maternal diabetes, including pre-pregnancy diabetes and GDM, and the risk of CHD in offspring. This finding provides important information for preventing and detecting CHD in offspring.

## Declarations

### Availability of data and material

Data are unsuitable for public deposition due to ethical restrictions and legal framework of Japan. It is prohibited by the Act on the Protection of Personal Information (Act No. 57 of 30 May 2003, amendment on 9 September 2015) to publicly deposit the data containing personal information. Ethical Guidelines for Medical and Health Research Involving Human Subjects enforced by the Japan Ministry of Education, Culture, Sports, Science and Technology and the Ministry of Health, Labour and Welfare also restricts the open sharing of the epidemiologic data. All inquiries about access to data should be sent to: jecs-en@nies.go.jp. The person responsible for handling enquiries sent to this e-mail address is Dr Shoji F. Nakayama, JECS Programme Office, National Institute for Environmental Studies.
